# Oncolytic Maraba virus armed with tumor antigen boosts vaccine priming and reveals diverse therapeutic response patterns when combined with checkpoint blockade in ovarian cancer

**DOI:** 10.1186/s40425-019-0641-x

**Published:** 2019-07-17

**Authors:** A. J. Robert McGray, Ruea-Yea Huang, Sebastiano Battaglia, Cheryl Eppolito, Anthony Miliotto, Kyle B. Stephenson, Amit A. Lugade, Gill Webster, Brian D. Lichty, Mukund Seshadri, Danuta Kozbor, Kunle Odunsi

**Affiliations:** 1 Center for Immunotherapy, Roswell Park Comprehensive Cancer Center, Elm and Carlton Sts, Buffalo, NY 14263 USA; 2 McMaster Immunology Research Centre, McMaster University, Hamilton, ON Canada; 3 Turnstone Biologics, Ottawa, ON Canada; 4 Innate Immunotherapeutics, Auckland, NZ New Zealand; 5 Department of Dentistry and Maxillofacial Prosthetics, Roswell Park Comprehensive Cancer Center, Buffalo, NY USA; 6 Department of Immunology, Roswell Park Comprehensive Cancer Center, Buffalo, NY USA

**Keywords:** Ovarian cancer, Oncolytic virus, Cancer vaccine, Cancer, Immunotherapy, Adaptive immune resistance, PD-1 blockade, MRI

## Abstract

**Background:**

Cancer immunotherapies are emerging as promising treatment strategies for ovarian cancer patients that experience disease relapse following first line therapy. As such, identifying strategies to bolster anti-tumor immunity and limit immune suppression, while recognizing diverse patterns of tumor response to immunotherapy is critical to selecting treatment combinations that lead to durable therapeutic benefit.

**Methods:**

Using a pre-clinical mouse model, we evaluated a heterologous prime/boost vaccine in combination with checkpoint blockade to treat metastatic intraperitoneal ovarian cancer. Vaccine-elicited CD8^+^ T cell responses and changes in the tumor microenvironment following treatment were analyzed and compared to treatment outcome. Kinetics of intraperitoneal tumor growth were assessed using non-invasive magnetic resonance imaging (MRI).

**Results:**

Vaccine priming followed by antigen-armed oncolytic Maraba virus boosting elicited robust tumor-specific CD8^+^ T cell responses that improved tumor control and led to unique immunological changes in the tumor, including a signature that correlated with improved clinical outcome of ovarian cancer patients. However, this treatment was not curative and T cells in the tumor microenvironment (TME) were functionally suppressed. Combination PD-1 blockade partially overcame the adaptive resistance in the tumor observed in response to prime/boost vaccination, restoring CD8^+^ T cell function in the TME and enhancing the therapeutic response. Non-invasive MRI of tumors during the course of combination treatment revealed heterogeneous radiologic response patterns following treatment, including pseudo-progression, which was associated with improved tumor control prior to relapse.

**Conclusions:**

Our findings point to a key hierarchical role for PD-1 signaling and adaptive immune resistance in the ovarian TME in determining the functional fate of tumor-specific CD8^+^ T cells, even in the context of robust therapy mediated anti-tumor immunity, as well as the ability of multiple unique patterns of therapeutic response to result in durable tumor control.

**Electronic supplementary material:**

The online version of this article (10.1186/s40425-019-0641-x) contains supplementary material, which is available to authorized users.

## Background

Epithelial ovarian cancer (EOC) accounts for 140,000 deaths annually worldwide and is the leading cause of gynecologic cancer-related mortality in the United States [1–3]. Although improved clinical outcome in ovarian cancer has been associated with increased intraepithelial CD3^+^ and/or CD8^+^ tumor infiltrating lymphocytes (TILs) [4–6], vaccine strategies aimed at expanding tumor-specific T cells in ovarian cancer patients have demonstrated modest clinical responses [7–9]. Similarly, while immune checkpoint inhibitors have generated remarkable results in several tumor types (e.g. melanoma, NSCLC, bladder cancer) leading to FDA approval, the response rates in EOC are lower (~ 5–10%) [10, 11]. A major barrier to successful cancer immunotherapy is the low tumor mutation burden [12] and the immunosuppressive microenvironment (TME) of ovarian cancer. Even if large numbers of tumor-specific T cells are generated therapeutically, these T cells may not readily destroy tumor targets in vivo because they encounter (i) a suppressive milieu that protects tumor cells from immune destruction (“innate immune resistance”); and (ii) counter-regulatory adaptation to tumor-specific immune responses (“adaptive immune resistance”) [13, 14]. As a result, efforts to improve or restore anti-tumor immunity by reprogramming the TME to overcome multiple immunosuppressive pathways are highly desirable [15, 16]. However, a major gap in ovarian cancer remains a lack of understanding of the optimal context(s) for generating or restoring tumor immune attack by vaccination or blocking checkpoint receptors [17].

Oncolytic viruses (OV) directly target tumor cells for destruction, while also promoting inflammation in the TME and anti-tumor immune responses [18, 19]. OVs induce in situ vaccination against tumor antigens as they are released in the inflamed TME and taken up by DCs, eliciting T cell immunity against the entire tumor antigen repertoire. Therefore, the subsequent epitope spreading has the potential to serve as a personalized immunotherapy and convert immunologically inert tumors, including those with low mutation burden such as EOC, into highly immune-reactive ones. In line with these preclinical observations, advanced melanoma patients treated with an engineered herpes virus (talimogene laherparepvec, T-Vec) developed melanoma antigen-specific (MART-1) T cells within injected and non-injected lesions [20], suggesting that local OV injection induces potent systemic antitumor immunity. Unfortunately, OV clinical trials in ovarian cancer have been less successful. A randomized phase IIB trial of single-agent weekly paclitaxel compared with weekly paclitaxel plus reovirus Serotype 3 (GOG 186-H), showed similar median PFS (4.3 mo vs 4.4 mo) and OS (13.1 mo vs 12.6 mo) in both arms [21]. In clinical trials of i.p. administration of oncolytic measles virus engineered to express carcinoembryonic antigen (MV-CEA) [22] or sodium iodide symporter (MV-NIS) [23] for recurrent EOC, the best objective response was stable disease in 14/21 and 13/16 patients, respectively. We reasoned that the relatively low mutation burden of ovarian cancer and consequent limited repertoire of tumor neoantigens likely contribute to the lack of efficacy of OVs.

To address these issues, we investigated the capacity of oncolytic Maraba virus (MRB) [24, 25] “armed” with a tumor antigen to enhance therapeutic vaccination by driving antigen specific T cells into the TME in an intraperitoneal metastatic murine ovarian cancer model. We also sought to understand kinetics of tumor response in the peritoneal microenvironment because complex and dynamic tumor response patterns have been observed across multiple cancers following immunotherapy [26, 27]. Whether unique tumor response features are indicative of early therapeutic efficacy, response durability, or treatment failure represents a gap in knowledge with important clinical implications. We employed a tumor antigen-specific heterologous prime/boost approach and observed that antigen-armed OV drives expansion of tumor antigen-specific T cells and concomitantly counteracts multiple immune suppressive elements. However, the immunological pressure exerted by the antigen-armed OV led to adaptive upregulation of the PD-1/PD-L1 axis and other inhibitory ligands as a means of self-protection, and these further contributed to immune resistance. Finally, non-invasive magnetic resonance imaging identified distinct radiologic response patterns following combination treatment with PD1 blockade, highlighting the complexity and diversity of therapeutic responses.

## Methods

### Cell culture

The ID8 cell line has been extensively used as a murine model of metastatic ovarian cancer [28]. To generate readily trackable biological effects within the tumor, we utilized IE9-mp1, a fast growing variant of IE9, that expresses the model antigen OVA and GFP, and has previously been described [17]. A luciferase expressing variant of ID8 (ID8-FLUC) was generated using a pFU-Luc2-Tomato lentiviral vector encoding firefly luciferase (FLUC) and td-Tomato. ID8, IE9-mp1, and ID8-FLUC cell lines were grown in complete RPMI (cRPMI) as detailed in Additional file 1: Supplemental methods. Cell lines were IMPACT tested prior to use in vivo. Vero Cells were purchased from ATCC and were grown in DMEM containing 10% FBS and 1% Pen/Strep.

### Mice

Female C57BL/6 J mice were purchased from the Jackson Laboratory (Bar Harbor, ME) and bred in the Roswell Park Comprehensive Cancer Center (RPCCC) animal facility under an established breeding protocol or were purchased directly from Jackson Laboratory prior to experimental use. OT-1 T cell receptor (TCR) transgenic mice were bred in the RPCCC animal facility, and were used as a source of OVA-specific OT-1 T cells for in vitro co-culture assays. Experimental mice were 6–8 weeks of age at study onset. All performed experiments and procedures have been reviewed and approved by the RPCCC IACUC.

### MIS416 + OVA vaccination and Maraba virus

MIS416 is a microparticle vaccine adjuvant derived from *Propionibacterium acnes* and comprised of immune-stimulatory muramyl dipeptide and bacterial DNA, which signals through NOD-2 and TLR9 receptors, and is capable of inducing DC maturation and cross-presentation that promotes CTL polarization and Th1 immunity [29]. For vaccine studies, MIS416 (550 μg/mouse) was mixed with ovalbumin protein (18 μg/mouse) and injected in 200 μl PBS split between two subcutaneous sites (both dorsally, between the scapulae and in line with the hind limbs) (MIS416 Vax). The attenuated strain MG1 of Maraba virus has been previously described [24, 30] and was used in all studies. Insertion of transgenes into MG1 vectors was between the G and L viral genes. Maraba-OVA expresses full-length ovalbumin (OVA) and Maraba-hDCT expresses the full-length human melanoma antigen dopachrome tautomerase (DCT) and was used as an irrelevant control vector where indicated (MRB-CONT). Recombinant Maraba virus was prepared and titered at McMaster University, shipped on dry ice to RPCCC, and stored at − 80 °C prior to use.

### In vitro oncolysis and virus titering from tumors

IE9-mp1 (1.5 × 10^4^) cells were plated in triplicate in 96 well plates and cultured overnight prior to Maraba virus infection. The next morning, media was removed and cells were infected at increasing multiplicity of infection (MOI, range 10^− 5^-10^1^ pfu/cell prepared in 20 μl of cRPMI) for 45 min at 37 °C. 180 μl of cRPMI was added to each well and cells were cultured for 24 h. Percent cell viability was assessed by MTT assay as described in Additional file 1: Supplemental methods. For titering of Maraba from mouse tissues, tissues were removed at indicated time points post infection (3 mice/treatment/time point) and snap frozen on dry ice in pre-weighed tubes containing PBS. The complete protocol is detailed in Additional file 1: Supplemental methods.

### Tumor challenge and immunization

Mice were challenged with 10^7^ IE9-mp1 or ID8-FLUC cells in 500 μl PBS by IP injection. For IE9-mp1 studies, mice were immunized with MIS416 Vax as described beginning 5 or 12 days post tumor implantation. MIS416 Vax was delivered twice at 5 day intervals with Maraba boosting occurring 10 days following the first MIS416 Vax prime. In preliminary studies, dosing of 10^8^–10^9^ pfu/mouse was tested to determine maximum tolerated Maraba dose that did not produce toxicity (data not shown). 2–4 × 10^8^ pfu Maraba was generally well tolerated and was used in all in vivo studies. Maraba was delivered by either intraperitoneal (IP), intravenous (IV), or split dose (IV/IP) injection. Tumor progression was tracked based on increase in abdominal circumference due to accumulation of peritoneal ascites. Mice were euthanized when abdominal circumference was ≥10 cm or when mice exhibited reduced body condition due to tumor progression. For bioluminescence studies, ID8-FLUC tumor-bearing mice were injected IP with 200 μl of 15 μg/μl D-Luciferin Potassium Salt (Gold Biotechnology, St Louis, MO) prepared in PBS and were imaged using an IVIS Spectrum and data analyzed using Living Image Software (Perkin Elmer, Waltham, MA). Data are reported as relative change in total photon flux (p/s) compared to baseline as a measure of changes in tumor burden in response to therapy.

### Monitoring of T cell responses

Blood, spleen, and peritoneal lavage (collected following IP injection of PBS) were collected at indicated time points to monitor tumor-specific CD8^+^ T cell responses. Red blood cells were removed from prepared single cell suspensions using ACK lysis buffer and CD8^+^ T cell responses to the immunodominant epitope of ovalbumin (OVA_257–264_; SIINFEKL) were measured by tetramer staining. For direct ex vivo analysis of CD8^+^ T cell function based on cytokine production, tissue single cell suspensions were stimulated with SIINFEKL peptide (1 μg/ml) for 5 h in the presence of Brefeldin A (10 μg/ml) (Sigma Aldrich, St Louis, MO). CD8^+^ T cell reactivity to parental ID8 cells (epitope spreading experiments) is detailed in Additional file 1: Supplemental methods. Data were acquired using a LSR II flow cytometer (BD Biosciences) and analyzed using FlowJo software (TreeStar, Ashland, OR).

### Monoclonal antibodies and flow cytometry staining

Monoclonal antibodies were delivered to mice by IP injection (200 μg/mouse in 200 μl of PBS). For depletion experiments, anti-CD8α antibodies (clone 2.43) were purchased from Bio X Cell (West Lebanon, NH). Anti-PD-1 (clone RMP1–14) was purchased from Bio X Cell (West Lebanon, NH). Delivery schedules are detailed in Additional file 1: Supplemental Methods. Flow cytometry antibodies were purchased from BD Biosciences (San Jose, CA), eBiosciences (Thermo Fisher Scientific, Waltham, MA), or BioLegend (San Diego, CA) and clones/staining procedures are detailed in Additional file 1: Supplemental Methods.

### Immunohistochemistry

Solid tumor nodules from the omentum along with surrounding tissue were excised from mice and fixed in 10% Neutral Buffered Formalin for a period of 3 days and were processed, sectioned, and stained at the RPCCC Pathology Resource Network using Agilent Technologies/products (Santa Clara, CA), as detailed in Additional file 1: Supplemental methods. Slide images were scanned using Aperio Digital Pathology slide scanner and analyzed and scored using ImageScope software (Leica Biosystems, Buffalo Grove, IL) by an independent Pathologist at RPCCC.

### RNA isolation from solid tumors, Nanostring data analysis, and integration of TCGA data

Solid tumor nodules were identified and carefully excised from the omentum of mice 15 days following treatment onset and were snap frozen on dry ice and RNA prepared as detailed in Additional file 1: Supplemental methods. Nanostring analysis was then carried out (4 biological replicates/treatment) using the RPCCC Genomics Shared Resource. Samples were run in groups of twelve samples according to the manufacturer’s specifications (Nanostring Technologies, Seattle WA) with at least 2 technical replicates/sample using the Mouse PanCancer Immune reporter code set (Cat # XT-GXA-MIP1–12). Data normalization and analysis was performed using nSolver Software version 2.6. To determine the gene signature associated with MIS416 Vax + MRB-OVA (prime/boost signature), raw Nanostring data were quality controlled, processed, and normalized via geometric mean utilizing the nSolver Analysis software. Normalized data were then imported into R [31] and voom-transformed with the limma package [32]. Differentially expressed genes unique to this therapy were identified via ANOVA and post-hoc Tukey correction and only genes that significantly changed following this treatment combination were deemed part of the prime/boost signature. To assess the clinical significance of the prime/boost signature using patient data from TCGA, RNASeq and clinical data from ovarian cancer patients were downloaded as median Zscores from cBioportal [33]. Gene expression for the “prime/boost signature” were extracted for all patients and clustered utilizing affinity propagation clustering (APCluster) [34], out of which we identified three main patient clusters. Survival analysis was performed with the R survival package [35].

### Culture of primary tumor explants and analysis of OVA antigen expression

Once mice had reached experimental endpoint, solid tumor nodules were excised and finely minced in cRPMI. The resulting tumor slurry was plated and cultured in cRPMI for 48 h prior to thorough washing, at which point growing cell monolayers were identified. Cells were allowed to grow until reaching 80–90% confluence and were visually confirmed to have similar morphology to the IE9-mp1 cell line. Primary explant cultures were then passaged once prior to use. Detection of OVA expression by Western Blot or via OT-1 T cell recognition assays are detailed in Additional file 1: Supplemental Methods.

### Magnetic resonance imaging (MRI)

MRI examinations of mice was performed using a 4.7-T/33-cm horizontal bore magnet (GE NMR Instruments, Fremont, CA) incorporating a removable gradient coil insert (G060; Bruker Medical Inc., Billerica, Mass) generating maximum field strength of 950 mT/m and a custom-designed 35-mm RF transmit-receive coil. All animal procedures and tumor volume calculations from MRI analysis have been detailed in the Additional file 1: Supplemental methods.

### Statistical analysis

Two-tailed, unpaired t tests were used to compare data from two treatment groups. One and two way Analysis of Variances (ANOVA) were used for data analysis of more than two groups and a Bonferroni post-test was utilized to determine significant differences between groups. Survival data was compared using a Logrank test. Results were generated using GraphPad Prism software. Differences between means were considered significant at *p* < 0.05: * *p* < 0.05, ** *p* < 0.01, *** *p* < 0.001. NS: not significant.

## Results

### Tumor antigen armed oncolytic Maraba virus directly targets ovarian tumors while acting as a booster vaccine

**Fig. 1:**
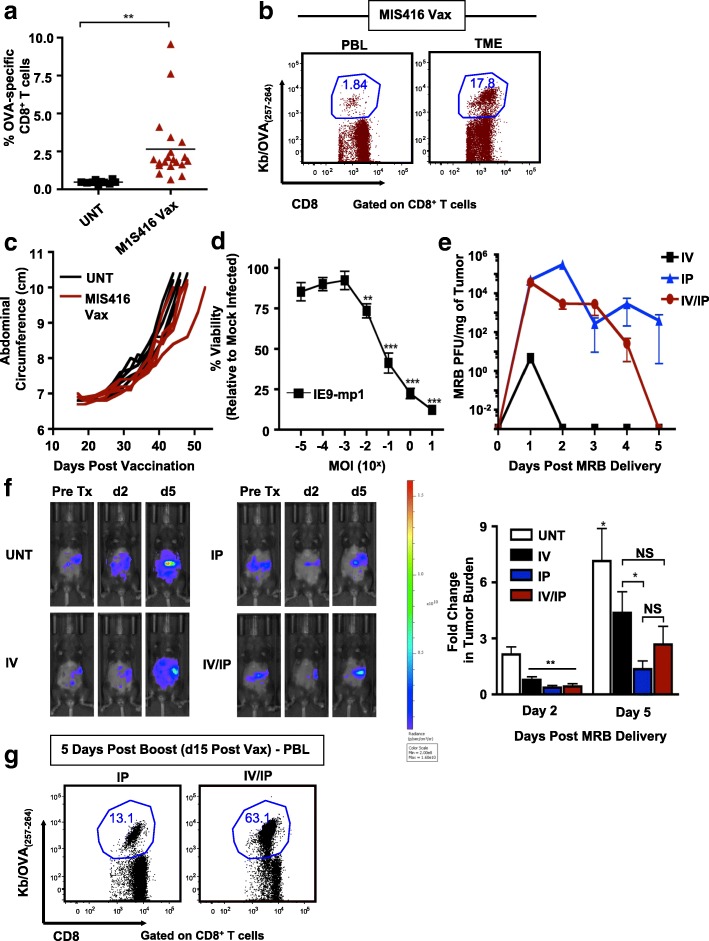
Maraba delivery targets ovarian tumors for oncolysis and boosts vaccine-elicited anti-tumor T cell responses. **a** OVA-specific CD8^+^ T cells were assessed in the blood of untreated (■) or MIS416 Vax treated () mice on d10 post vaccination (*n* = 10–20). **b** Representative FACs plots from a single mouse treated with MIS416 Vax showing **%** OVA-specific CD8^+^ T cells in the PBL and peritoneal TME **c** IE9-mp1 tumor progression was assessed based on increasing abdominal circumference of mice following vaccination (*n* = 5). **d** IE9-mp1 cells were infected with MRB at increasing MOI and cell viability assessed 24 h post infection. **e** Titer of replicating MRB virus in tumor tissue over time following IV (■), IP (), or IV/IP () virus delivery (*n* = 3 mice/group/time point). **f** Tumor load was assessed by bioluminescent imaging of ID8-FLUC tumor-bearing mice at indicated time points following virus delivery (*n* = 4–5). **g** Representative FACs plots depicting OVA-specific CD8^+^ T cell responses combining MIS416 Vax with MRB-OVA boosting by different routes. Data presented as mean ± SEM. Data in **c** is from one representative experiment and (**d**) compiled from 3 independent experiments

Employing an aggressive murine ovarian cancer model engineered to express OVA (IE9-mp1) [17], we tested whether the endogenous OVA-specific T cell response elicited through vaccination could be therapeutic. Mice bearing 5 day intraperitoneal (IP) IE9-mp1 tumors were immunized with OVA admixed with MIS416, a non-toxic microparticle adjuvant derived from *Propionibacterium acnes* [29] (MIS416 Vax). Given that MIS416 signals through NOD-2 and TLR9, and has been previously shown to induce DC maturation, production of inflammatory cytokines, and antigen cross-presentation, leading to expansion of antigen-specific T cells when delivered along with target antigen [29], we reasoned that MIS416 Vax would elicit a potent OVA-specific T cell response, leading to anti-tumor immunity. Vaccination generated modest circulating OVA-specific CD8^+^ T cell responses (Fig. 1a), with preferential trafficking of tumor-specific CD8^+^ T cells into the TME (Fig. 1b) but did not improve tumor progression over untreated animals (Fig. 1c).

We reasoned that rapid tumor growth combined with inadequate anti-tumor T cell responses prevented therapeutic efficacy. We therefore sought to identify strategies to concomitantly promote an immunogenic TME, enhance direct tumor cell killing, and also amplify vaccine-elicited T cell responses. In this regard, oncolytic Maraba virus (MRB) can effectively target ovarian cancer cells [24, 36] and in vitro testing revealed IE9-mp1 to be highly sensitive to MRB-mediated oncolysis (Fig. 1d).

To determine the optimal delivery route for targeting intraperitoneal (IP) ovarian tumors, we examined IP, intravenous [IV], or split dose injection [IV/IP] and found both increased peak intratumoral viral titer and persistence by IP injection, followed by IV/IP, with limited transient virus detected following systemic IV delivery (Fig. 1e). MRB delivery by all routes reduced tumor burden 2 days post-delivery compared to baseline (Fig. 1f). However, by day 5, IP treatment showed a significant improvement in tumor control compared to IV, with IV/IP having an intermediate effect (Fig. 1f), demonstrating a clear advantage to direct virus delivery into the IP tumor site to mediate oncolysis.

MRB has previously been shown to elicit only weak T cell responses when used as a priming vaccine, but functions as a robust vaccine booster [30]. Therefore, we tested whether delivery of antigen-armed MRB (MRB-OVA) could enhance MIS416 Vax primed T cell responses when delivered either IP or IV/IP. Systemic delivery was required to achieve maximal vaccine boosting, with IV/IP MRB-OVA generating > 4-fold expansion of circulating OVA-specific CD8^+^ T cells compared to IP delivery at the same dose (Fig. 1g). We therefore reasoned that split dose IV/IP delivery of MRB provided the best strategy to promote oncolysis of IP ovarian tumors while also boosting MIS416 Vax responses and was used in subsequent therapeutic studies.

### Oncolytic Maraba boosting following vaccination slows the progression of metastatic ovarian cancer

**Fig. 2:**
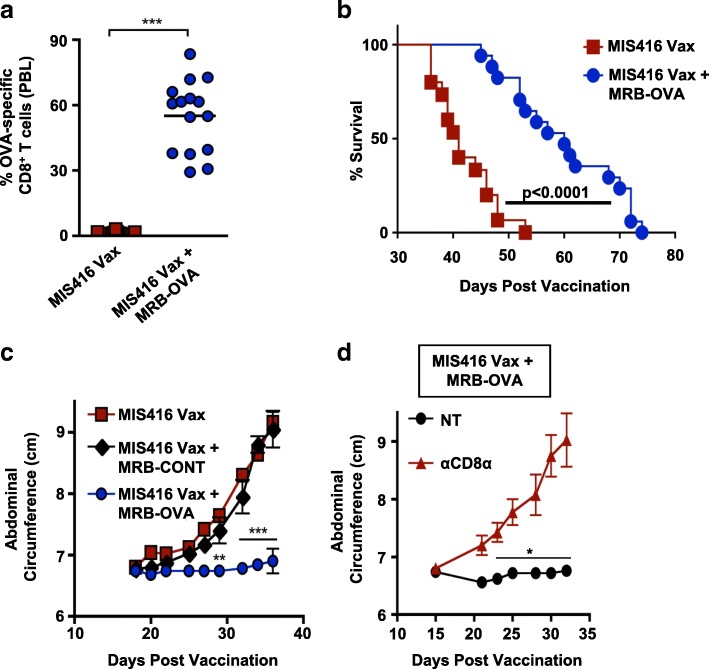
Heterologous prime/boost vaccination elicits dramatic expansion of tumor-specific CD8^+^ T cells and slows tumor progression. **a** % OVA-specific CD8^+^ T cells was assessed in the blood on d15 following MIS416 Vax () or MIS416 Vax + MRB-OVA () (*n* = 15). **b** Compiled survival data of d5 tumor-bearing mice following MIS416 Vax () or MIS416 Vax + MRB-OVA () (*n* = 15–17). **c** Tumor progression in mice following MIS416 Vax alone () or in combination with MRB-CONT (♦) or MRB-OVA () (*n* = 4–5). **d** Tumor progression in mice following MIS416 Vax + MRB-OVA alone (NT, ●) or in combination with CD8α depletion (anti-CD8α,) (*n* = 4–5). Data presented as mean ± SEM. Data in **c** and **d** are from one representative experiment

We next tested whether boosting with MRB could alter tumor progression and survival following vaccination. To allow sufficient time between vaccine priming and MRB boosting [30], we first tested combination therapy using a day 5 therapeutic model (Additional file 2: Figure S1a). MIS416 Vax followed by MRB-OVA boosting led to significant expansion of OVA-specific CD8^+^ T cells compared to vaccination alone (Fig. 2a), which persisted at high frequency in circulation (Additional file 2: Figure S1b), and significantly improved survival of tumor-bearing mice (Fig. 2b). In contrast, delivery of control MRB expressing the irrelevant transgene hDCT (MRB-CONT) did not delay tumor progression beyond vaccination alone (Fig. 2c), indicating improved tumor control following MRB delivery was antigen dependent and required antigen expression directly from MRB virus. Consistent with these data, depletion of CD8^+^ T cells using an anti-CD8α antibody abrogated tumor control following MRB-OVA boosting, confirming a CD8^+^ T cell dependent mechanism (Fig. 2d).

### Boosting with antigen-armed MRB increases tumor-specific CD8^+^ T cell TME infiltration, but does not prevent local T cell suppression

**Fig. 3:**
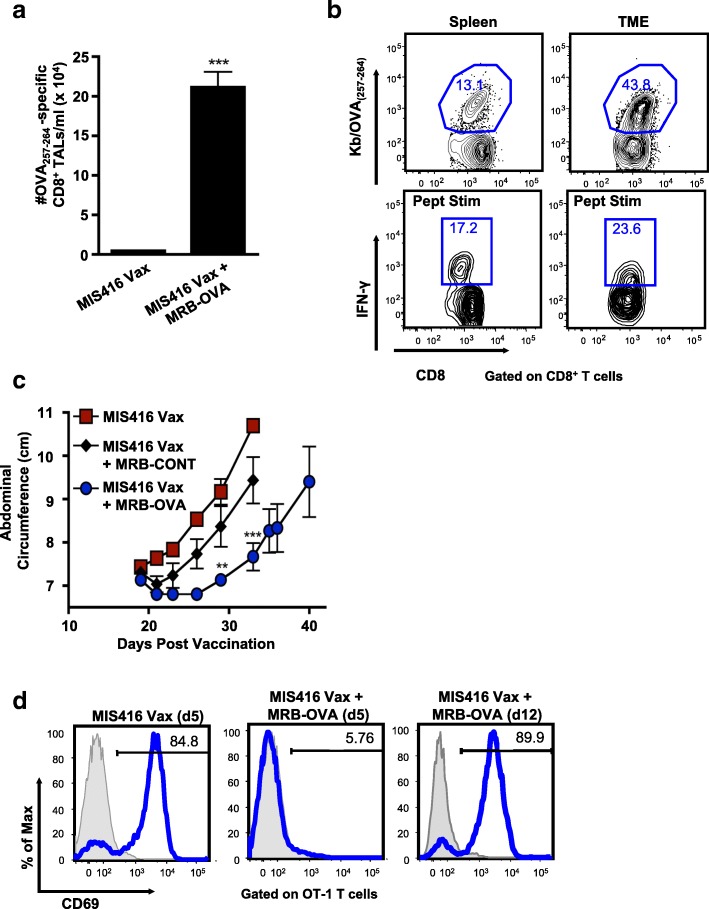
Maraba boosting alters the inflammatory tumor microenvironment, however tumors escape immune clearance via multiple mechanisms. **a** OVA-specific CD8^+^ T cells were enumerated in the peritoneal TME on d15 post therapy onset (*n* = 7). **b** Representative FACs plots depicting % OVA-specific CD8^+^ T cells by tetramer staining and corresponding IFN-γ production following ex vivo OVA_257–264_ peptide stimulation in matched spleen and TME samples on d15 following MIS416 Vax + MRB-OVA. **c** IE9-mp1 tumor progression in mice using a d12 therapeutic model following MIS416 Vax alone () or in combination with MRB-CONT (♦) or MRB-OVA () (*n* = 3). **d** Representative FACs plots measuring OT-1 T cell activation (based on CD69 upregulation) following co-culture with IE9-mp1 tumor explants collected at endpoint (blue histograms). Gray histograms show CD69 surface expression on naïve OT-1 cells cultured alone in parallel. Data presented as mean ± SEM

Although MIS416 Vax + MRB-OVA improved tumor control, tumor-bearing mice ultimately progressed. To investigate mechanisms of immune escape, we analyzed the OVA-specific CD8^+^ T cells in the TME. Five days after MRB boosting (day 15 post vaccination), we found increased OVA-specific CD8^+^ T cells within the peritoneal lavage (tumor associated lymphocytes, TALs) following prime/boost therapy compared to vaccination alone (Fig. 3a). As expected, the frequency of OVA-specific CD8^+^ T cells was higher in the TME compared with spleen following prime/boost therapy (Fig. 3b). However, OVA-specific CD8^+^ T cells had decreased functionality in the TME when compared to the spleen, having a reduced ratio of IFN-γ^+^ to tetramer^+^CD8^+^ T cells (Fig. 3b & Additional file 3: Figure S2a), as well as diminished IFN-γ production by functional cells (Additional file 3: Figure S2b).

Even with reduced functionality compared to peripheral cells, the number of functional tumor-specific CD8^+^ TALs was not consistent with a complete lack of curative treatment. Interrogation of endpoint tumor explants revealed that tumors isolated from untreated and MIS416 Vax treated animals continued to express OVA, while MRB-OVA boosting resulted in outgrowth of OVA-negative tumors consistent with antigen loss (Additional file 3: Figure S2c). Somewhat surprisingly, tumor antigen loss variants (ALV) were also observed following IP administered MRB-OVA boost, where the OVA-specific CD8^+^ T cell response was considerably lower than by IV/IP delivery, suggesting the anti-tumor T cell response following IV/IP boosting was well beyond the threshold required to eliminate all OVA-expressing tumor cells in the day 5 therapeutic model.

We next questioned whether treatment of more established tumors would also result in outgrowth of ALV. Treatment was delayed until 12 days post tumor implantation and while MIS416 Vax + MRB-OVA (IV/IP) delayed tumor progression compared to MIS416 Vax ± MRB-CONT, the duration of treatment efficacy was reduced compared to the day 5 model (Fig. 3c; median survival of 48 days and 60 days for MIS416 Vax + MRB-OVA in the day 12 and day 5 models, respectively). Importantly, while naïve OVA-specific OT-1 T cells were not activated (based on CD69 upregulation) following co-culture with endpoint tumor explants derived from day 5 MIS416 Vax + MRB-OVA treated mice (consistent with ALV), OT-1 T cells were readily activated by co-culture with endpoint tumor explants from day 12 treated tumors (MIS416 Vax + MRB-OVA), indicating continued target antigen expression/presentation (Fig. 3d). Taken together, these data suggest that while tumor escape following prime/boost therapy can be driven by antigen loss when tumor burden is low, more established tumors continue to express target antigen and progress under conditions of significant anti-tumor immunity, but reduced T cell function within the TME.

### Tumor immune profiling reveals a unique gene signature of prime/boost therapy that correlates with clinical outcome

**Fig. 4:**
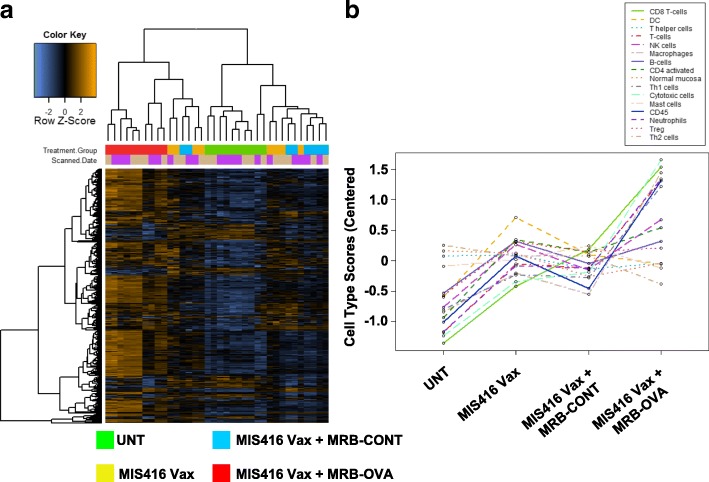
Tumor immune profiling reveals a unique gene signature of tumor targeted prime/boost therapy **a** Hierarchical cluster analysis of intratumoral transcriptional changes between treatment group (FDR < 0.1) (*n* = 8–10). **b** Immune cell profiling across treatment group (*n* = 8–10). All analysis was performed using the nCounter Immune Profiling Advanced Analysis plugin for nSolver

**Fig. 5:**
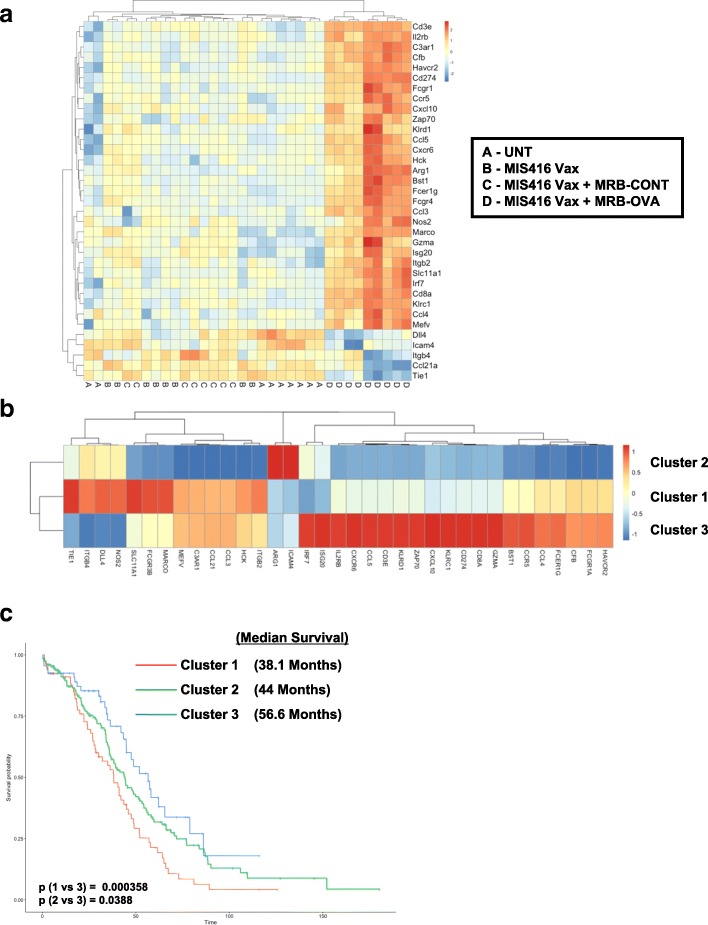
“Prime/boost” gene signature from MIS416 Vax + MRB-OVA mapped to ovarian cancer patients from TCGA. **a** Heat map depicting unique 35 gene signature identified in MIS416 Vax + MRB-OVA treated tumors (*n* = 8–9). **b** Hierarchical clustering of ovarian cancer patients from TCGA based on expression of genes identified in **a** (*n* = 307). **c** Ovarian cancer patient survival based on individual clusters identified in **b**

We reasoned that investigating the local TME might point to immunological mechanisms limiting CD8^+^ T cell function and therapeutic efficacy of prime/boost therapy. To this end, we used the day 12 therapeutic model to perform whole tumor immune profiling by Nanostring. Solid IP tumors were isolated 15 days following treatment onset (corresponding to the peak T cell response observed following prime/boost therapy, Additional file 2: Figure S1b) from untreated animals, or mice treated with MIS416 Vax ± MRB-CONT or MRB-OVA. Hierarchical clustering revealed a unique gene signature associated with MIS416 Vax + MRB-OVA (Fig. 4a). Immune cell profiling using nSolver software suggested that intratumoral changes following MIS416 Vax ± MRB-CONT were consistent with an altered local immune landscape compared to untreated tumors. However, MIS416 Vax + MRB-OVA was associated with greater accumulation of CD45^+^ immune cells, including CD8^+^/Cytotoxic T cells, macrophages, neutrophils, Th1 polarized cells, and to a lesser extent NK cells and activated CD4^+^ T cells (Fig. 4b). Further analysis led to identification of a 35 gene “prime/boost” signature unique to only MIS416 Vax + MRB-OVA therapy (Fig. 5a) that, when mapped to publicly available ovarian cancer patient data from TCGA (*n* = 307 patients), separated patients into 3 distinct clusters (Fig. 5b), one of which correlated with improved clinical outcome (Fig. 5c). Notably, the major gene subset uniquely associated with this cluster (Cluster 3) was consistent with a CD8^+^ T cell signature/local T cell function, in line with findings from the mouse model.

### Prime/boost therapy is limited by adaptive resistance via the PD-1/PD-L1 axis and can be improved through checkpoint blockade

**Fig. 6:**
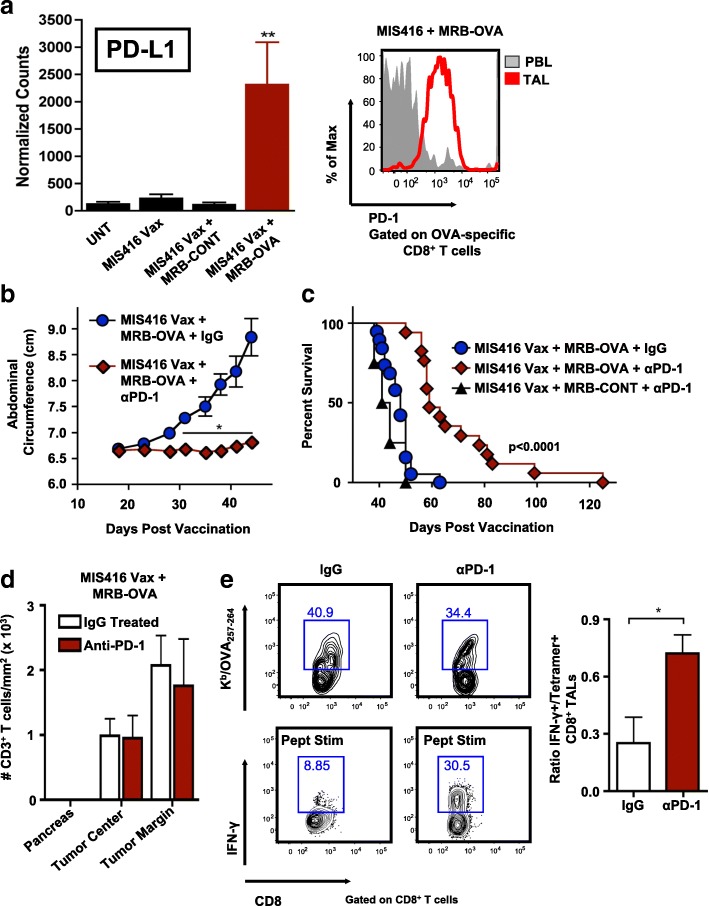
Prime/boost therapy is dramatically improved via PD-1 blockade through reversal of tumor-specific T cell dysfunction*.* **a Left:** Intratumoral expression of PD-L1 assessed by Nanostring as described in Fig. 4 (*n* = 8). **Right:** Representative FACS data showing PD-1 expression on OVA-specific CD8^+^ T cells in the blood (PBL) or TME (TAL). **b** Day 12 IE9-mp1 tumor progression in mice following MIS416 Vax + MRB-OVA combined with IgG () or anti-PD-1 () (*n* = 8–9). **c** Compiled survival data of day 12 tumor-bearing mice following MIS416 Vax + MRB-CONT + anti-PD-1 (▲) or MIS416 Vax + MRB-OVA + IgG () or anti-PD-1 () (*n* = 4–19). **d)** CD3^+^ T cell infiltration was enumerated at either the tumor center or margins following MIS416 Vax + MRB-OVA + IgG or anti-PD-1. Adjacent pancreas was poorly infiltrated and served as an indicator of specific T cell trafficking to tumors. (*n* = 4). **e Left Panel:** Representative FACs plots depicting % OVA-specific CD8^+^ T cells by tetramer staining and corresponding IFN-γ production following ex vivo OVA_257–264_ peptide stimulation in matched TME samples on day 25 following MIS416 + Vax + MRB-OVA + IgG or anti-PD-1 treatment. **Right Panel:** OVA-specific CD8^+^ TAL function was assessed based on ratio of % IFN-γ producing to tetramer^+^CD8^+^ T cells as shown (*n* = 4). Data presented as mean ± SEM. Data in **b** is from one representative experiment

MIS416 Vax + MRB-OVA resulted in significant transcriptional changes associated with T cells (Additional file 4: Figure S3 & Additional file 7: Table S1), including elevated expression of co-inhibitory and/or co-stimulatory pathways. We noted increased expression of PD-L1 (CD274) in solid tumors, consistent with adaptive immune resistance, as well as increased expression of PD-1 on tumor-specific CD8^+^ TALS following MIS416 Vax + MRB-OVA (Fig. 6a, and Additional file 7: Table S1). We reasoned that blockade of PD-1 might improve OVA-specific CD8^+^ TAL function within the TME, amplifying the impact of prime/boost therapy. Mice bearing 12 day IE9-mp1 tumors were immunized with MIS416 Vax + MRB-OVA, with anti-PD-1 or IgG control antibody treatment commencing on the day of boosting. Combining anti-PD-1 with prime/boost vaccination significantly delayed peritoneal ascites development and extended survival compared to prime/boost therapy alone in an antigen-specific manner (Fig. 6b & c). Adding PD-1 blockade to MIS416 Vax + MRB-OVA did not impact the peripheral T cell response (Additional file 5: Figure S4a), but trended towards increasing the number of OVA-specific CD8^+^ TALs (Additional file 5: Figure S4b). Importantly, while the number of CD3^+^ TILs at either the tumor center or margin was not affected by anti-PD-1 (Fig. 6d and Additional file 5: Figure S4c), prime/boost-elicited OVA-specific CD8+ TALs produced significantly more IFN-γ following PD-1 blockade in response to ex vivo peptide stimulation (Fig. 6e), demonstrating that improved therapeutic efficacy was driven by enhanced T cell function and not simply increased TIL/TAL number. Analysis of endpoint tumor explants revealed outgrowth of OVA-negative tumors in 33% of MIS Vax + MRB-OVA + anti-PD-1 treated mice (data not shown), suggesting that development of ALV prevented determination of full therapeutic potential. Notably, low-level recognition of OVA-negative parental ID8 cells by CD8^+^ T cells isolated from the spleen following MIS416 Vax + MRB-OVA + anti-PD-1 indicated antigen spreading to non-OVA antigen(s)/epitope(s) following treatment (Additional file 5: Figure S4d), however these non-OVA targeted responses were not sufficient to mediate durable cure of any animals.

### Non-invasive imaging reveals diverse response patterns following prime/boost + anti-PD-1 therapy

**Fig. 7:**
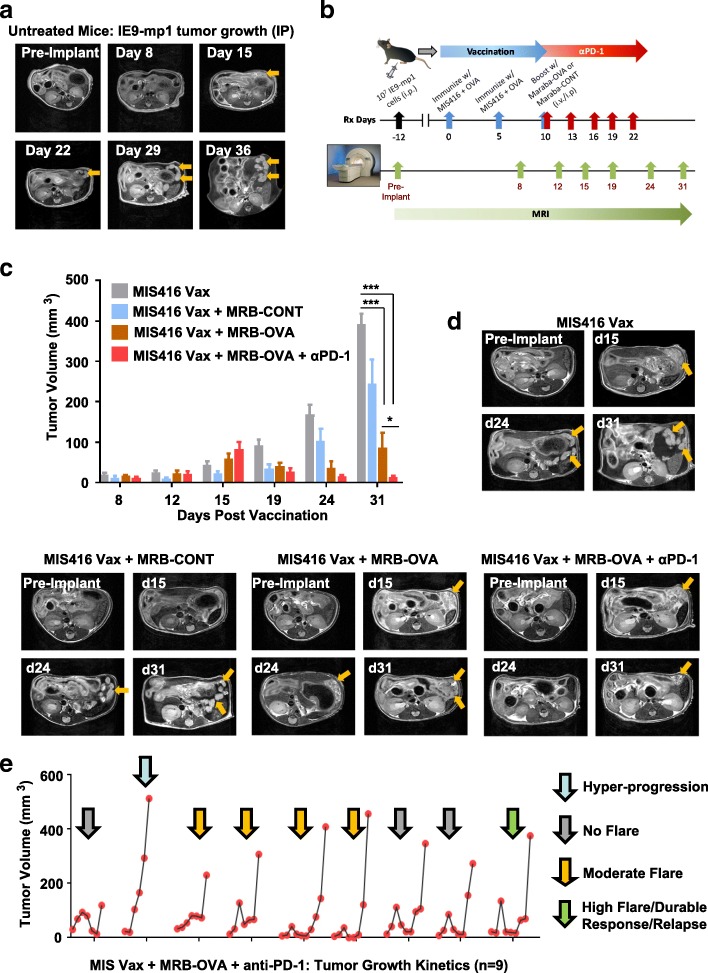
MRI reveals improved tumor control and distinct response patterns following antigen-targeted prime/boost vaccination + anti-PD-1. **a** Axial T2-weighted images of a mouse over time demonstrating onset and growth of tumor lesions (yellow arrows) following IE9-mp1 tumor implantation (*n* = 5). **b** Schematic representation of study design for serial monitoring of response to combination therapy **c** MR-based tumor volume measurements of peritoneal lesions in mice from control and treatment groups at different times post vaccination (*n* = 3–5). **d** Axial T2-weighted images of a representative animal from all 4 experimental groups illustrating differences in tumor growth kinetics. Tumor lesions indicated with yellow arrows. **e** Temporal changes in the tumor volume of individual animals (*n* = 9) treated with MIS Vax + MRB-OVA + anti-PD-1 illustrating the heterogeneity in response pattern. Data presented as mean ± SEM

Monitoring accumulation of ascites is a surrogate measure of tumor progression in the IP IE9-mp1 model, but does not allow direct assessment of tumor response to therapy. To understand the kinetics of tumor response in the peritoneal cavity, we utilized non-invasive MRI to longitudinally monitor disease progression/therapeutic response. Studies in untreated mice revealed that IE9-mp1 tumors initially seeded in the omentum of injected animals (Fig. 7a), with distinct tumor nodules emerging after approximately 2 weeks. Growth of the primary tumor lesion was evident in later scans, along with onset of ascites.

To test whether tumor response to therapy could be monitored in a similar way, we used the day 12 therapeutic IE9-mp1 model in the context of vaccination (MIS416 Vax), OV (MRB-CONT or MRB-OVA), and checkpoint blockade (anti-PD-1) (Fig. 7b). MIS416 Vax + MRB-CONT demonstrated only modest improvement in tumor control over MIS416 Vax (Fig. 7c & d), with MIS416 Vax + MRB-OVA resulting in marked tumor regression followed by relapse. Consistent with our previous data, inclusion of anti-PD-1 in combination with MIS416 Vax + MRB-OVA further increased the degree of tumor regression and response duration compared to targeted prime/boost therapy (Fig. 7c & d). Unexpectedly, imaging of animals on day 15 post vaccination (corresponding to the peak T cell response following prime/boost therapy; Additional file 2: Figure S1b) revealed that mice treated with MIS416 Vax + MRB-OVA ± anti-PD-1 (orange and red bars) had increased tumor volume compared to MIS416 Vax (gray bars) and MIS416 Vax + MRB-CONT (blue bars) (Fig. 7c). However, subsequent scans revealed continued tumor growth in MIS Vax ± MRB-CONT, but tumor regression in both MIS416 Vax + MRB-OVA ± anti-PD-1, with maximal tumor growth inhibition observed with anti-PD-1 treatment (Fig. 7c & d), consistent with pseudo-progression following OVA-targeted prime/boost therapy.

We questioned whether the pseudo-progression or transient ‘flare’ in tumor volume prior to regression was an early indicator of the degree or durability of therapeutic response. To address this, we focused on MIS416 Vax + MRB-OVA + anti-PD-1 treatment, where the tumor volume flare and improved therapeutic response were best demonstrated. Evaluation of changes in tumor volume by MRI revealed 4 distinct therapeutic response patterns, ranging from no flare (3/9), moderate flare (4/9), high flare (1/9), and hyper-progression (1/9) (Fig. 7e). Interestingly, the degree of pseudo-progression at day 15 post vaccination did not correlate with the duration of therapeutic efficacy (Additional file 6: Figure S5a), with both dramatic tumor regression following pseudo-progression and durable stable disease both demonstrating similar long term responses (Additional file 6: Figure S5b). Together, these data suggest that distinct response patterns to MIS Vax + MRB-OVA + anti-PD-1 can produce similar therapeutic outcomes, and that pseudo-progression alone is not predictive of improved therapeutic response.

## Discussion

Using a metastatic ovarian cancer model, oncolytic Maraba virus armed with tumor antigen efficiently enhanced therapeutic vaccination. However, durable tumor control was limited by induction of immunosuppressive elements in response to therapy, in particular signaling via PD-1 to T cells. We further demonstrated that the improved efficacy of the armed oncolytic MRB following PD-1 blockade was accompanied by enhanced T cell function and not significant changes in TIL/TAL number as a result of checkpoint blockade. The observation that TAL function could be recovered in a subset of OVA-specific CD8^+^ TALs following PD-1 blockade suggests these TALs developed the recently described plastic or re-programmable dysfunctional state as opposed to a fixed dysfunctional state [37], although the specific chromatin states or surface markers reportedly associated with plastic versus fixed dysfunctional states were not specifically evaluated in our study. As multiple immunosuppressive factors were expressed in tumors following prime/boost therapy, including PD-L1/PD-L2, Arginase 1 and 2, NOS2, and additional checkpoint receptors (CTLA-4, LAG3, TIM3, and TIGIT) (Additional file 8: Table S2), it is highly probable that blocking or inhibiting these additional suppressive pathways (either alone or using combinatorial strategies) could also influence the functional fate of T cells within the TME following prime/boost vaccination and formally testing such combinations warrants further investigation in follow up studies.

Through ongoing efforts, cancer vaccines continue to show clinical promise, with cancer patients being treated using a variety of vaccine platforms and antigen targeting strategies based on new and emerging knowledge [38–42]. Included among these approaches are four clinical trials testing oncolytic Maraba virus in the context of vaccine boosting (NCT02285816, NCT02879760, NCT03618953, NCT03773744; 3 active, 1 not yet recruiting). In the present study, the potential clinical significance of boosting vaccine primed T cells with an antigen armed oncolytic virus was demonstrated by analyzing the “prime/boost” signature in the ovarian cancer TCGA cohort. Consistent with the preclinical data, patients with elevated expression of “prime/boost” signature genes associated with T cell infiltration/function demonstrated improved survival, supporting the importance of strategies aimed at generating potent anti-tumor T cell responses. While the robust T cell responses reported herein were generated against an immunogenic model antigen, detection of impressive spontaneous TIL responses to tumor neoantigens has been reported [43] and suggests that generating potent anti-tumor T cell responses through vaccination may be feasible in the context of immunogenic target antigen(s). In light of this, previously vaccinated ovarian cancer patients or those with measurable anti-tumor T cell responses could benefit from MRB boosting by targeting relevant antigens/epitopes.

Using longitudinal imaging of solid IP tumor lesions, we observed pseudo-progression in the majority of animals following targeted prime/boost therapy ± anti-PD-1. In contrast, while pseudo-progression has been observed in patients responding to checkpoint inhibitors, the incidence is typically low [26, 44, 45]. Given the inflammatory TME generated through prime/boost therapy in this model and the high frequency of pseudo-progression observed, it is probable that increased inflammation within the TME improves the likelihood of observing a pseudo-progression event. Importantly, our data indicate that the same therapeutic regimen can produce additional response patterns that led to durable tumor control in the context of robust anti-tumor immunity. Conversely, rapid tumor progression following immunotherapy (hyper-progression, also seen in our study) has been observed in a small subset of patients [46, 47], suggesting that the relationship between dynamic changes in tumor size and ultimate therapeutic response is complex. Kinetic analysis of the TME using transcriptomics/proteomics over the course of MIS416 Vax + MRB-OVA + anti-PD-1 is currently underway by our group and may provide additional insights as to how the anti-tumor immune response/immune landscape in treated tumors varies over time and across the diverse patterns of response observed following therapy.

Checkpoint inhibitors, including anti-PD-1, have shown remarkable clinical activity in subsets of patients across tumor indications and ongoing efforts to identify characteristics of a patient’s tumor and/or immune status that are likely to predict response to checkpoint blockade continue to be aggressively pursued [48]. However, checkpoint monotherapies have shown limited efficacy in ovarian cancer patients [11, 49] and combination therapies incorporating available checkpoint inhibitors are being evaluated clinically [50]. Combining OV with checkpoint blockade has shown promise in both pre-clinical cancer models [51, 52] and early clinical trials [20, 53, 54]. However, these studies did not utilize antigen armed OV (oncolytic vaccines), which we observed to dramatically improve PD-1 blockade. As multiple strategies of arming OV are currently being explored pre-clinically and/or in clinical trials, including delivery of cytokines, chemokines, or immunostimulatory ligands [55], expression of checkpoint blocking agents directly from the OV [56, 57], or delivery of tumor antigens as an oncolytic vaccine as explored in this study, the optimal strategies and context for delivering armed OV in combination with checkpoint blockade to cancer patients remains to be identified. Importantly, we did note toxicity in a subset of MIS416 Vax + MRB-OVA treated animals that received anti-PD-1 therapy (5 out of more than 50 mice), which was not observed following MRB-CONT boosting. These data suggest that toxicities associated with checkpoint inhibitors may be exacerbated in the context of heightened T cell responses, especially when localized to the peritoneum, even when T cells are targeting tumor restricted antigens and will thus require careful consideration when such strategies are employed clinically.

In the current study, outgrowth of tumor ALV was a mechanism of immune escape following prime/boost therapy. While ALV were not observed following OVA-targeted prime/boost therapy in the more advanced disease setting, addition of anti-PD-1 resulted in ALV in a subset of treated tumors. As all IE9-mp1 cells were killed following 72 hour co-culture with OVA-specific OT-1 T cells (data not shown), it does not appear that the starting cell population harbors readily detectable OVA-negative variants, but that these variants emerge through immunological pressure. While the ability to elicit T cell responses that can effectively eliminate all antigen expressing tumor targets is encouraging, this observation has key implications in the context of single antigen targeting, given the likelihood of heterogeneous tumor antigen expression and evidence for both single and multi-antigen loss in recent clinical trials [58–61]. It is noteworthy that IE9-mp1 is a polyclonal pool of OVA-expressing cells, thus possessing some attributes of tumor heterogeneity. Additional studies using a cloned cell population (where ALV would presumably not arise) in the context of prime/boost vaccination could help to elucidate whether tumors are completely cleared in the absence of ALV outgrowth or whether additional (potentially novel) mechanisms of therapeutic resistance emerge.

Although the use of OVA as a model antigen could be considered a limitation of our study and may increase the probability of ALV emerging, the low but detectable CD8^+^ T cell reactivity against the OVA-negative parental ID8 cell line following OVA-targeting prime/boost + anti-PD-1 therapy provides indirect evidence of antigen/epitope spreading that may prolong immune attack in the context of ALV. Given that ID8 has a low mutational load that does not give significant rise to bona fide neo-epitopes effectively presented to T cells [62], these low-level responses could be targeting endogenous antigens. However, whether T cell responses to these additional antigens are due to tumor antigen release in the immunogenic context of MRB-driven oncolysis, as we have observed previously when employing an oncolytic vaccinia virus expressing a CXCR4 antagonist [63], or through direct tumor attack by prime/boost vaccine-elicited T cells is unclear. Additionally, some frequency of virus-specific T cells is likely to be generated as a result of the current prime/boost strategy [64] and these T cells may play some role in promoting local inflammation within the TME as a result of the anti-viral response. Lastly, while we have focused on monitoring CD8^+^ T cell responses to the immunodominant OVA epitope (OVA_257–264_; SIINFEKL), given that the prime/boost vaccine targets the full OVA antigen (in addition to OVA antigen released from IE9-mp1 cells), it is possible that CD8^+^ T cell responses specific for additional OVA epitopes, including the newly identified CD8^+^ T cell epitopes reported by Karandikar et al [65], are generated and may contribute to tumor attack. Whether these T cells, potentially targeting multiple tumor antigens/epitopes, can be expanded to ultimately sustain tumor attack is currently under investigation and will improve our understanding of the full breadth of the CD8^+^ T cell response generated through prime/boost vaccination and how to best apply such approaches clinically.

## Conclusion

Our findings demonstrate the potent effects of heterologous prime/boost vaccination incorporating antigen-armed oncolytic viruses and the value of this approach for treating metastatic ovarian cancer. We show that such an approach may be limited by adaptive immunosuppression in the TME acting on T cells, particularly PD-1 signaling, that prevent durable tumor control. Additionally, robust anti-tumor immunity driven by prime/boost therapy can lead to multiple therapeutic response patterns when combined with checkpoint blockade (including pseudo-progression) that are associated with improved response durability, highlighting a need to understand the complex dynamics of the TME when evaluating responses to combination immunotherapies.

## Additional files

Additional file 1: Supplemental Methods. (DOCX 24 kb)

Additional file 2: **Figure S1.** Tumor-specific CD8^+^ T cells expand and persist following prime/boost therapy. **a**) Schematic representation of experimental design and treatment schedule. **b**) % OVA-specific CD8^+^ T cells was measured in the blood following MIS416 Vax + MRB-OVA treatment (*n* = 5–15). d15 data reproduced from Fig. 2a for reference. Data presented as mean ± SEM. (PDF 122 kb)

Additional file 3: **Figure S2.** OVA-specific CD8^+^ T cell responses following prime/boost therapy and development of ALV post therapy. ***a***) Ratio of IFN-γ^+^ to OVA tetramer^+^CD8^+^ T cells was determined in spleen and TME following MIS416 Vax + MRB-OVA (*n* = 7). **b)** IFN-γ median fluorescent intensity (MFI) of IFN-γ^+^CD8^+^ T cells following ex vivo peptide stimulation (*n* = 7). **c)** Detection of OVA expression in primary IE9-mp1 endpoint tumor explants isolated following treatment (*n* = 3). Parental IE9-mp1 cell line was included as a positive control. (PDF 178 kb)

Additional file 4: **Figure S3.** Volcano plot showing differentially expressed genes associated with T cell function in tumors comparing MIS416 Vax + MRB-OVA to untreated animals (*n* = 8–10). All analysis was performed using the nCounter Immune Profiling Advanced Analysis plugin for nSolver. (PDF 264 kb)

Additional file 5: **Figure S4**. *Changes in the OVA-specific CD8*^*+*^ *T cell response following MIS416 Vax + MRB-OVA ± anti-PD-1.* **a)** % OVA-specific CD8^+^ T cells in the blood following MIS416 Vax + MRB-OVA + IgG (■) or αPD-1 (▲) or MIS416 Vax + MRB-CONT + anti-PD-1 (▼) (*n* = 5–14). **b)** OVA-specific CD8^+^ TALs were enumerated in the TME following MIS416 Vax + MRB-OVA + IgG or anti-PD-1 (*n* = 4). **c**) Representative CD3 staining of tumors isolated from mice following treatment with MIS416 Vax + MRB-OVA + IgG or anti-PD-1. Scale bar = 400 μm. **d**) FACS plots depicting reactivity against parental ID8 cells (OVA-negative) based on IFN-γ production by CD8^+^ T cells isolated from a long lived MIS416 Vax + MRB-OVA + anti-PD-1 treated mouse compared to naïve control cells. Data presented as mean ± SEM. (PDF 539 kb)

Additional file 6: **Figure S5.** *Correlation between tumor response pattern and response durability following MIS Vax + MRB-OVA + anti-PD-1.* **a**) Correlation between the change in tumor volume at pseudo-progression ‘flare’ compared to previous scan and the time to disease endpoint (measured based on development of abdominal distension due to ascites accumulation requiring euthanasia as outlined in methods). **b)** Tumor growth curves from individual treated animals demonstrating both pseudo-progression followed by regression (left panel) or stable disease (right panel) prior to disease relapse (*n* = 9). (PDF 105 kb)

Additional file 7: **Table S1.** Differentially Expressed Genes MIS416 Vax + MRB-OVA vs Untreated. (XLS 26 kb)

Additional file 8: **Table S2.** Upregulated Immunosuppressive Genes: MIS416 Vax + MRB-OVA vs Untreated. (XLS 23 kb)

## Data Availability

The datasets used and/or analyzed during the current study are available from the corresponding author on reasonable request.

## Abbreviations

ALV: Antigen loss variants
ANOVA: Analysis of variance
DCT: Dopachrome tautomerase
IP: Intraperitoneal
IV: Intravenous
MIS416 Vax: MIS416 + OVA
MOI: Multiplicity of infection
MRB: Maraba virus
MRI: Magnetic resonance imaging
OV: Oncolytic viruses
OVA: Ovalbumin
TALs: Tumor associated lymphocytes
TCR: T cell receptor
TILs: Tumor infiltrating lymphocytes
TME: Tumor microenvironment
T-VEC: Talimogene laherparepvec
