# Supplementary material for: Oncolytic Maraba virus armed with tumor antigen boosts vaccine priming and reveals diverse therapeutic response patterns when combined with checkpoint blockade in ovarian cancer
Source: J Immunother Cancer. 2019 Jul 17;7:189. doi: 10.1186/s40425-019-0641-x (PMC6637574; doi:10.1186/s40425-019-0641-x)
Supplement: Supplementary file 1 — Supplemental Methods. (DOCX 24 kb) [file 40425_2019_641_MOESM1_ESM.docx]

**Supplemental Methods**

*Culture media*

cRPMI media contains 10% FBS, 25mM Hepes, 2mM L-Glutamine, 100 IU/ml Pen/Strep, 1mM Sodium Pyruvate, 1x Non-Essential Amino Acids, and 0.05mM β-Mercaptoethanol.

*MTT assay*

20 μl MTT solution (5 mg/ml, prepared in H_2_O) was added to wells and incubated for 3 hrs at 37°C. Media was removed and 100μl DMSO added to wells. Plates were incubated at room temperature for 20 mins, followed by absorbance measurement at 570 nm using a spectrophotometer. Background (media only wells)-subtracted mean absorbances from infected cultures were compared to mock infected wells.

*Flow cytometry – antibodies and staining protocols*

The following antibodies were used in the reported cell staining (clones in parentheses): CD45-V450 (30-F11), CD8-FITC and CD8-PerCP (53-6.7), PD-1-PE (J43), CD69-APC (H1.2F3), IFN-γ-APC (XMG1.2). All antibodies were titrated for optimal staining for 30 min at 4°C. K^b^/OVA_257-264_-APC tetramer for monitoring OVA-specific CD8^+^ T cells was purchased from Baylor College of Medicine MHC Tetramer Production Core (Houston, TX). BD Fc Block (2.4G2) was used to inhibit non-specific antibody binding prior to staining (15 min at 4°C). For intracellular cytokine staining, cells were stained using the BD Cytofix/Cytoperm buffer kit following the manufacturers’ recommended protocols. For all studies of spleen/peritoneal wash (and accompanying blood samples), LIVE/DEAD fixable Near-IR (Thermo Fisher Scientific, Waltham, MA) staining was performed to ensure interrogation of only viable cells in downstream data analysis.

*Immunohistochemistry*

Formalin-fixed paraffin embedded sections were cut at 4 μm, placed on charged slides, and dried at 60°C for 1hr. Slides were cooled to room temperature and added to a Dako Omnis autostainer, were deparaffinized with Clearify, and rehydrated using graded alcohols. Flex TRS High pH was used for target retrieval for 30 min. Slides were incubated with anti-CD3 antibody (1:125) for 30 min at 1:125. Rabbit Envision+/HRP, polymer was applied for 30 min, followed by DAB chromogen for 5 mins for visualization. Slides were counterstained with Hematoxylin for 8 min then put into water. Slides were removed from the Omnis, cleared, and cover slipped.

*Western blot analysis of OVA expression*

For detection of OVA expression, cells were harvested and lysed in RIPA buffer [25 mM Tris pH 7.4, 150 mM NaCl, 0.1 % SDS, 0.5% sodium deoycholate 1% Triton X-100, 1 mM PMSF, and protease inhibitor cocktail (Roche)] on ice for 30 min, followed by centrifugation at 14000 rpm. 40 μg of cleared lysate was heated at 75°C (10 mins) before separation on a 12.5% SDS polyacrylamide gel and transfer to a PVDF membrane. Membranes were blocked for 1 hr in 5% non-fat dry milk in Tris-based saline buffer (10 mM Tris, pH 7.5, 150 mM NaCl, 0.1% Tween [TBST]), probed with ant-OVA (1:1000, Abcam) for 1 hr, washed with TBST and incubated with anti-rabbit secondary antibody (1:2000, KPL) for 1 hr. Signal was detected with enhanced chemiluminescence (Amersham/GE) and visualized using a ChemiDoc Imaging Systems (BIORAD).

*OT-1:tumor explant co-culture*

For T cell recognition assays, explant cultures were stimulated overnight with IFN-γ (100 ng/ml) to promote upregulation of MHC Class I, then 1-2×10^4^ tumor cells were plated in 96 well plates and cultured with naïve OT-1 T cells at a 20:1 tumor cell:T cell ratio. 48 hrs later, T cell activation was assessed by flow cytometry based on upregulation of the early activation marker CD69. Culture of OT-1 cells alone and/or with parental ID8 cells and addition of SIINFEKL peptide to OT-1 cultures and/or culture with the IE9-mp1 cell line served as negative and positive controls, respectively.

*RNA extraction*

RNA isolation was carried out by first homogenizing tumors in Trizol (Thermo Fisher Scientific, Waltham, MA) using 3 mm glass beads in a mini beadbeater (Biospec Products, Bartlesville, OK). Following phenol chloroform extraction, sample aqueous phase was further purified on RNeasy columns (Qiagen Inc, Valencia, CA) according to the recommended protocol and DNase treated using DNase I (Thermo Fisher Scientific, Waltham, MA) according to the manufacturer’s protocol to remove contaminating genomic DNA. RNA quality was measured on an Agilent Bioanalyzer (Santa Clara, CA) and samples with RIN score >4.0 were selected for downstream analysis by Nanostring.

*Detection of epitope spreading following prime/boost therapy*

CD8^+^ T cells from the spleens of endpoint animals were enriched by negative selection using the EasySep Mouse CD8^+^ T cell isolation kit (Stem Cell Technologies, Vancouver, Canada). To remove the majority of OVA-specific CD8^+^ T cells, cells were subjected to FACs sorting on a FACSAria II (BD Biosciences) to isolate K^b^/OVA_257-264_ tetramer negative CD8^+^ T cells. Isolated cells were co-cultured with IFN-γ stimulated ID8 cells (Non OVA-expressing parental line of IE9-mp1) at a 15:1 effector:target ratio for 2 hrs prior to addition of Brefeldin A for an additional 5hrs.

*Virus titering from tumors*

Following collection, tissues were weighed and homogenized using a variable speed Tissue-Tearor (Model 398, BioSpec Products). Cellular debris was pelleted by centrifugation (12 mins, 1500 rpm) and cleared supernatants used to prepare log dilutions of tissue homogenates (10^1^-10^5^). Dilutions were added to confluent Vero cell cultures (60 mm dishes) and incubated for 45 mins at 37°C (100 μl homogenate/plate, tested in duplicate), with rocking every 15 mins. Cells were then overlayed with 3 ml of 0.5% agarose prepared in MEM media + 10% FBS and once polymerized, plates were returned to cell incubator, and incubated at 37°C. Plaques were counted at 24 and 48 hrs and mean counts from duplicate plates between 10-100 were used to calculate PFU/mg of tissue.

*In vivo monoclonal antibody delivery*

CD8 depletion using an anti-CD8α antibody (clone 2.43) was commenced one day after Maraba-OVA boosting and was delivered on two consecutive days and then every third day for a total of 5 doses. Depletion of CD8^+^ T cells was confirmed to be greater than 98% in the peripheral blood by flow cytometry (data not shown). For PD-1 blockade, anti-PD-1 (Clone RMP1-14) was delivered every third day to mice beginning the day of Maraba boosting for a total of 5 doses.

*Magnetic resonance imaging*

Induction of anesthesia prior to imaging and maintenance during imaging was accomplished by inhalation of 2-3% isoflurane (Abbott Laboratories, Chicago, IL). Induction and maintenance of anesthesia during imaging were achieved by inhalation of 2% to 3% isoflurane in oxygen (Abbott Laboratories, Abbott Park, Ill). Anesthetized mice were placed in an MR-compatible ‘mouse sled’ (Dazai Research Instruments, Toronto, Canada) within a carrier tube and positioned in the scanner. The body tempera­ture of animals during image acquisition was maintained using an air heater system (SA Instruments Inc., Stony Brook, NY, USA) connected to a thermocouple embedded within the sled that provided feedback for temperature control. Preliminary localizer images were acquired to enable optimal slice prescription for T2-weighted scans. Multi-slice T2-weighted images were acquired at different times post injection of tumor cells into the peritoneal cavity as described previously (1,2). Following image acquisition, raw image sets were transferred to a processing workstation and processed using the medical imaging software, Analyze (version 10.0; AnalyzeDirect, Overland Park, KS). Peritoneal lesions were manually traced across slices and tumor volume was calculated by measuring the cross sectional area on each slice and multiplying their sum by the slice thickness.Tumor volume was calculated by measuring the cross-sectional area on each slice and multiplying their sum by the slice thickness. Disease ‘flare' and hyperprogression were defined by comparing the change in tumor volume (∆TV) from two MRI examinations obtained during the early treatment period to two MRI scans obtained during a pretreatment reference period similar to criteria utilized in clinical studies (3). In our mouse model, disease flare was defined as a greater increase in ∆TV during the on-treatment period compared the reference period: Minimum/No Flare (<2-fold increase in ∆TV), Moderate (2-5 fold increase in ∆TV), and High (>5-fold increase in ∆TV).

**References for Supplemental Methods**

1. Francescutti V, Rivera L, Seshadri M, Kim M, Haslinger M, Camoriano M, et al. The benefit of intraperitoneal chemotherapy for the treatment of colorectal carcinomatosis. Oncol Rep. 2013 Jul;30(1):35–42.

2. Bothwell KD, Shaurova T, Merzianu M, Suresh A, Kuriakose MA, Johnson CS, et al. Impact of Short-term 1,25-Dihydroxyvitamin D3 on the Chemopreventive Efficacy of Erlotinib against Oral Cancer. Cancer Prev Res (Phila). 2015 Sep;8(9):765–76.

3. Champiat S, Dercle L, Ammari S, Massard C, Hollebecque A, Postel-Vinay S, et al. Hyperprogressive Disease Is a New Pattern of Progression in Cancer Patients Treated by Anti-PD-1/PD-L1. Clinical Cancer Research. 2017 Apr 15;23(8):1920–8.
