# Supplementary figures and images for: Oncolytic Maraba virus armed with tumor antigen boosts vaccine priming and reveals diverse therapeutic response patterns when combined with checkpoint blockade in ovarian cancer
Source: J Immunother Cancer. 2019 Jul 17;7:189. doi: 10.1186/s40425-019-0641-x (PMC6637574; doi:10.1186/s40425-019-0641-x)

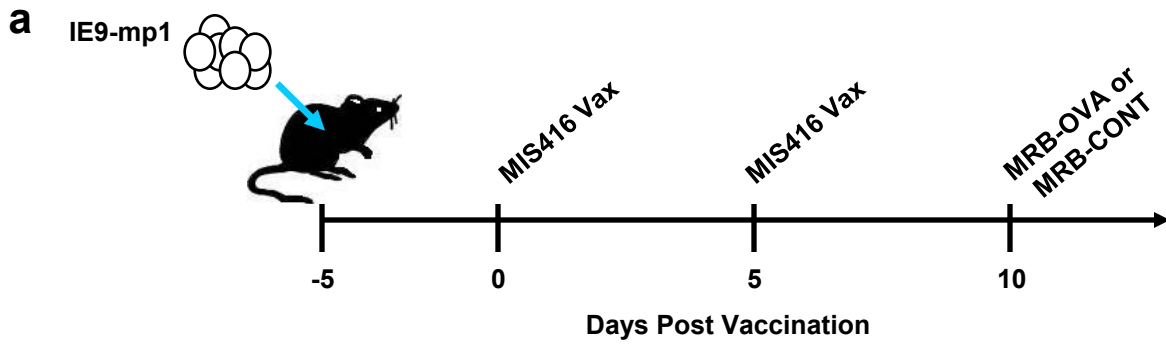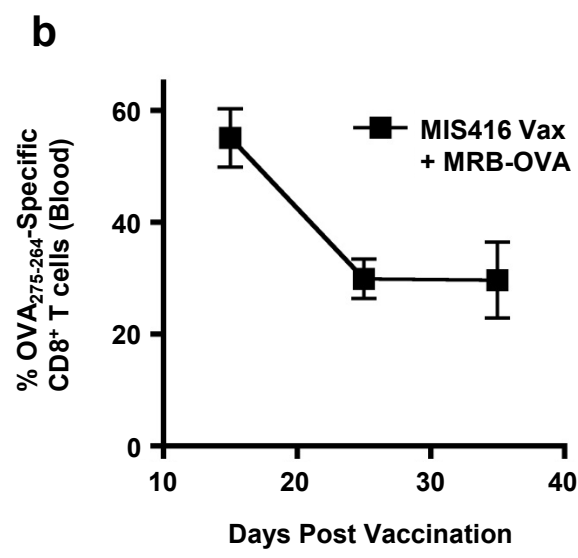

Supplement: Supplementary file 2 — Figure S1. Tumor-specific CD8+ T cells expand and persist following prime/boost therapy. a) Schematic representation of experimental design and treatment schedule. b) % OVA-specific CD8+ T cells was measured in the blood following MIS416 Vax + MRB-OVA treatment (n = 5–15). d15 data reproduced from Fig. 2a for reference. Data presented as mean ± SEM. (PDF 122 kb) [file 40425_2019_641_MOESM2_ESM.pdf]

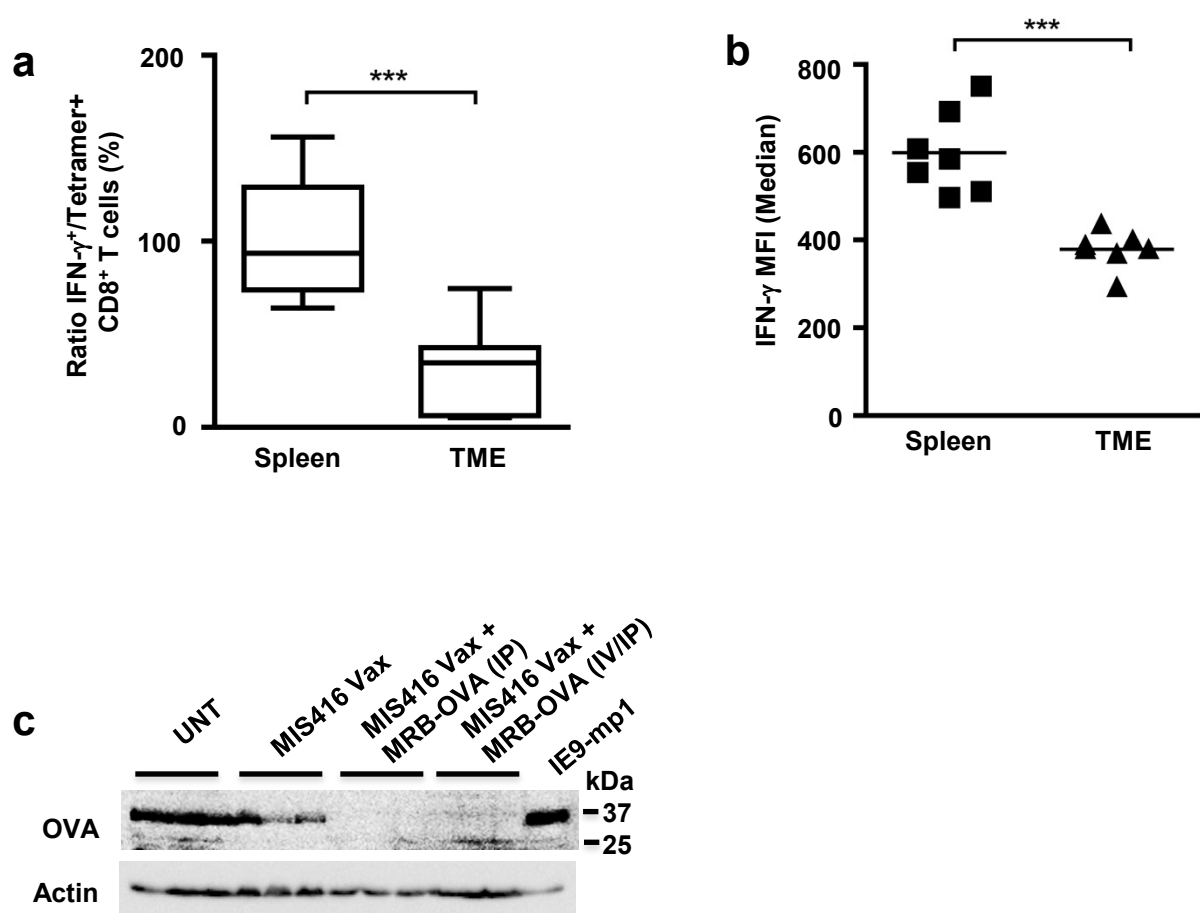

**Suppl. Fig 2**

Supplement: Supplementary file 3 — Figure S2. OVA-specific CD8+ T cell responses following prime/boost therapy and development of ALV post therapy. a) Ratio of IFN-γ+ to OVA tetramer+CD8+ T cells was determined in spleen and TME following MIS416 Vax + MRB-OVA (n = 7). b) IFN-γ median fluorescent intensity (MFI) of IFN-γ+CD8+ T cells following ex vivo peptide stimulation (n = 7). c) Detection of OVA expression in primary IE9-mp1 endpoint tumor explants isolated following treatment (n = 3). Parental IE9-mp1 cell line was included as a positive control. (PDF 178 kb) [file 40425_2019_641_MOESM3_ESM.pdf]

# MIS416 Vax + MRB-OVA vs UNT

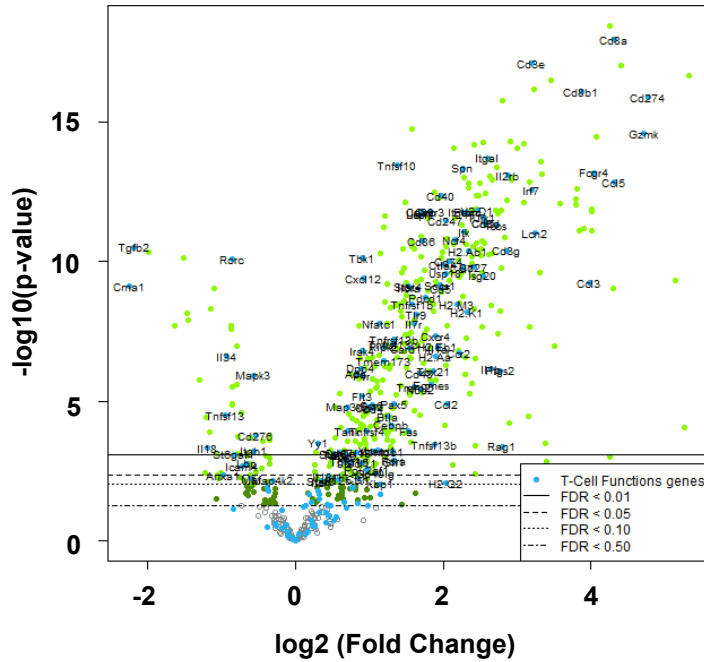

Supplement: Supplementary file 4 — Figure S3. Volcano plot showing differentially expressed genes associated with T cell function in tumors comparing MIS416 Vax + MRB-OVA to untreated animals (n = 8–10). All analysis was performed using the nCounter Immune Profiling Advanced Analysis plugin for nSolver. (PDF 264 kb) [file 40425_2019_641_MOESM4_ESM.pdf]

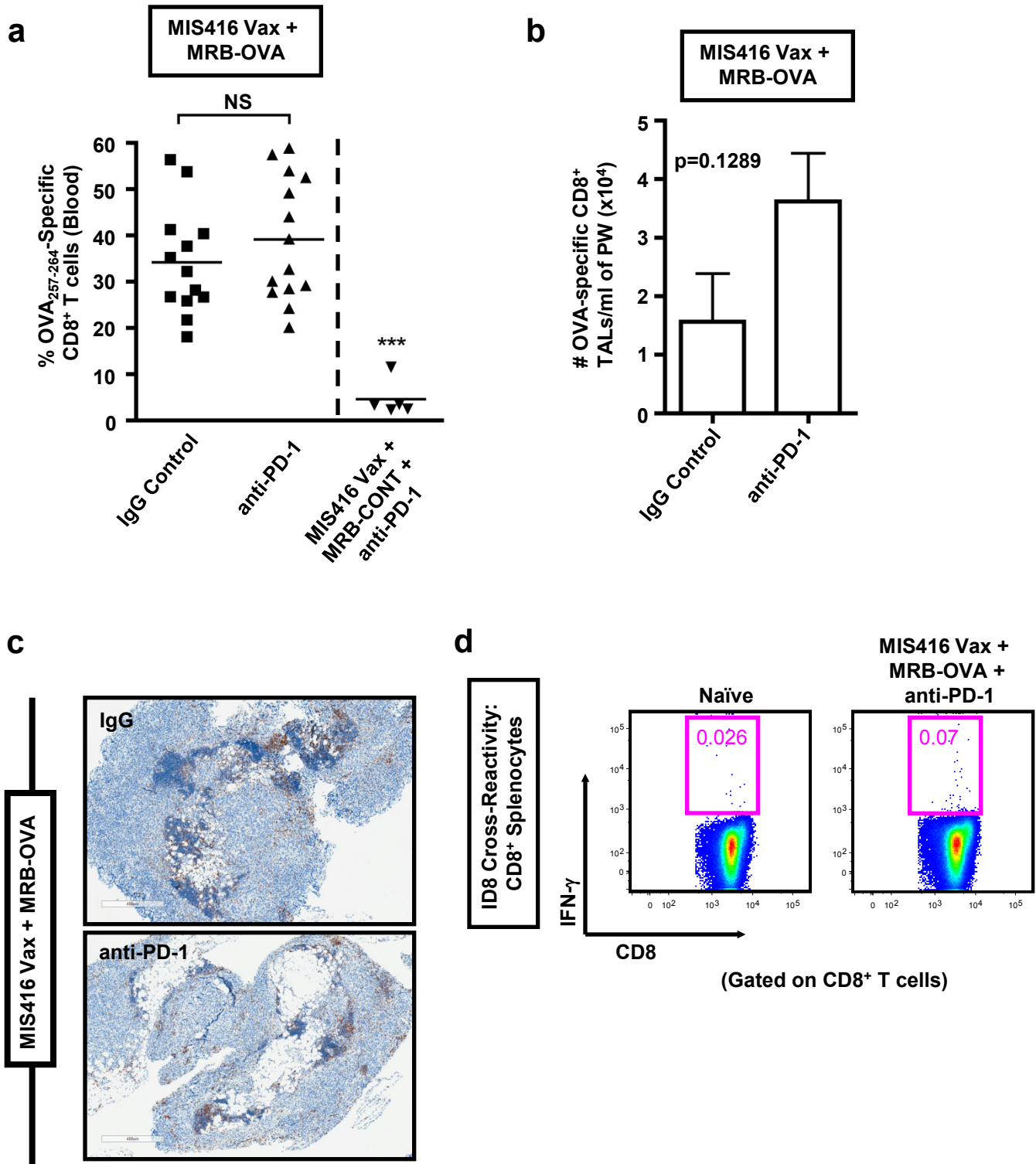

Suppl. Fig 4

Supplement: Supplementary file 5 — Figure S4. Changes in the OVA-specific CD8+ T cell response following MIS416 Vax + MRB-OVA ± anti-PD-1. a) % OVA-specific CD8+ T cells in the blood following MIS416 Vax + MRB-OVA + IgG (■) or αPD-1 (▲) or MIS416 Vax + MRB-CONT + anti-PD-1 (▼) (n = 5–14). b) OVA-specific CD8+ TALs were enumerated in the TME following MIS416 Vax + MRB-OVA + IgG or anti-PD-1 (n = 4). c) Representative CD3 staining of tumors isolated from mice following treatment with MIS416 Vax + MRB-OVA + IgG or anti-PD-1. Scale bar = 400 μm. d) FACS plots depicting reactivity against parental ID8 cells (OVA-negative) based on IFN-γ production by CD8+ T cells isolated from a long lived MIS416 Vax + MRB-OVA + anti-PD-1 treated mouse compared to naïve control cells. Data presented as mean ± SEM. (PDF 539 kb) [file 40425_2019_641_MOESM5_ESM.pdf]

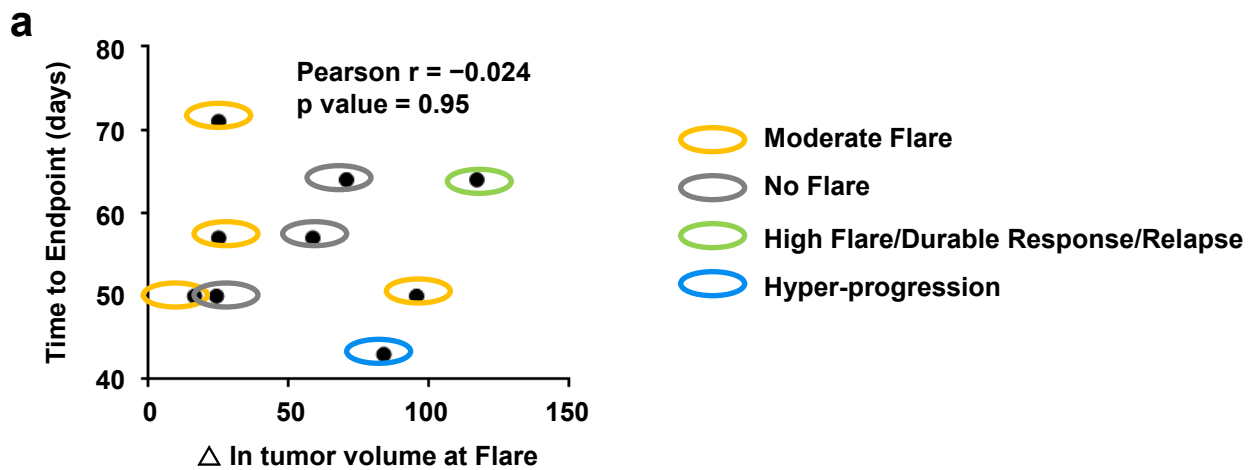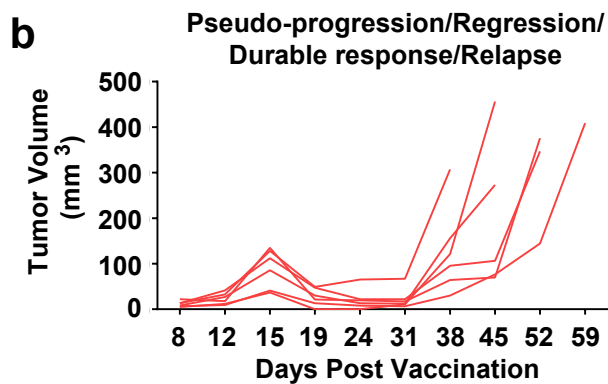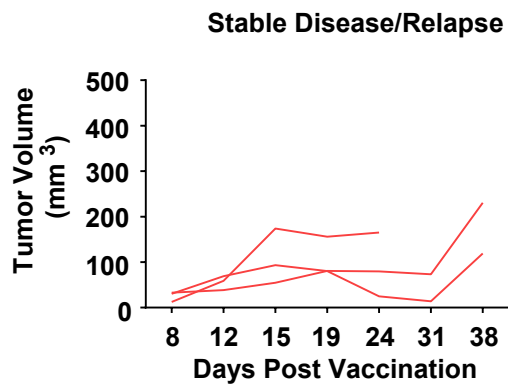

Supplement: Supplementary file 6 — Figure S5. Correlation between tumor response pattern and response durability following MIS Vax + MRB-OVA + anti-PD-1. a) Correlation between the change in tumor volume at pseudo-progression ‘flare’ compared to previous scan and the time to disease endpoint (measured based on development of abdominal distension due to ascites accumulation requiring euthanasia as outlined in methods). b) Tumor growth curves from individual treated animals demonstrating both pseudo-progression followed by regression (left panel) or stable disease (right panel) prior to disease relapse (n = 9). (PDF 105 kb) [file 40425_2019_641_MOESM6_ESM.pdf]
